# Vitamin E supplementation improves post-transportation systemic antioxidant capacity in yak

**DOI:** 10.1371/journal.pone.0278660

**Published:** 2022-12-02

**Authors:** Li Zhang, Zhiyu Wang, Peng Zhou, Lin Fu, Lijun Zhang, Changhui Xu, Juan J. Loor, Tao Zhang, Yi Chen, Ziyao Zhou, Xianwen Dong

**Affiliations:** 1 Chongqing Academy of Animal Sciences, Rongchang, China; 2 The Key Laboratory of Animal Disease and Human Health of Sichuan Province, College of Veterinary Medicine, Sichuan Agricultural University, Chengdu, China; 3 Tibet Leowuqi Animal Husbandry Station, Changdu Tibet, China; 4 Mammalian NutriPhysioGenomics, Department of Animal Sciences and Division of Nutritional Sciences, University of Illinois, Urbana, Champaign, IL, United States of America; 5 Chongqing Engineering Laboratory of Nano/Micro Biomedical Detection, School of Metallurgy and Materials Engineering, Chongqing University of Science and Technology, Chongqing, China; 6 Chongqing Key Laboratory of Nano/Micro Composite Material and Device, School of Metallurgy and Materials Engineering, Chongqing University of Science and Technology, Chongqing, China; The University of the West Indies, JAMAICA

## Abstract

This study was aimed to evaluate the effects of post-transportation vitamin E (VE) supplementation on health condition, blood biochemical parameters, blood antioxidant indices and blood metabolomics in yak. Five yaks were used in this study. After 2100 km of highway transportation from Riwoqe county to Rongchang County, Chongqing, blood was collected immediately after arrival and these samples served as the baseline (control, CON_VE). A VE injection (40 mg/kg) was then performed and blood samples were collected 10 days later. Injection of VE led to lower serum VE concentration. Relative to the CON_VE, VE injection led to greater concentrations of creatinine and lower concentrations of glutamate pyruvic transaminase, alkaline phosphatase, aspartate aminotransferase, total bilirubin, indirect bilirubin, direct bilirubin, UREA and glucose. Compared with CON_VE, VE injection led the lower serum level of malondialdehydeand greater serum level of glutathione s-transferase, glutathione peroxidase, glutathione reductase and glutathione peroxidase 4. Based on metabolomics analysis, 119 differentially altered serum metabolites (*P*<0.05 and VIP>1.0) were identified with VE injection relative to CON_VE. VE injection resulted in changes of lysophosphatidylethanolamine, lysophosphatidylcholine, phosphocholine, choline, malate, citrate, α-Oxo-glutarate, phenylalanine, 3-Phenylpropanoic acid and 3-(3-Hydroxyphenyl) propanoic acid. These metabolites are associated with lipid metabolism, tricarboxylic acid cycle and oxidative stress. Overall, our study indicates that VE injection can alleviate transportation stress in yak partly through protecting liver and kidney, and improving antioxidant defense systems.

## Introduction

Transportation stress refers to the instinctive adaptability and defensive response of the animal under the joint action of stress factors such as fasting, capture, vibration, collision, noise, crowding, scraping, environmental changes (density, temperature, humidity), and psychological pressure [1, 2]. It is one of the most important factors affecting animal production. The superposition of these stress factors leads to changes in body temperature [3], blood composition [4], meat quality, histopathological changes [5, 6], hormones, metabolites, and enzymes in the animal [1, 7–10]. Often, these changes have an adverse impact on production performance, immune level, animal husbandry product quality, animal welfare and health, and even lead to death [11, 12].

Stress factors have been shown to cause oxidative stress [13, 14], and stress factors lead to an imbalance between the production of free radicals and active metabolites (reactive oxygen species (ROS) or oxidants) and their elimination by protective mechanisms (antioxidants) [15]. This imbalance damages important biological macromolecules and cells, have a potential impact on the whole body [16], and eventually damage enzymes, proteins and lipids, leading to lipid peroxidation, protein carbonylation and the release of proinflammatory factors [17]. The accumulation of proinflammatory substances further promotes the formation of ROS and aggravates the stress condition [18] eventually causing cell death. Protecting the body from these harmful oxidants is a carried out by a complex system of non-enzymatic antioxidants (including vitamins E) and enzymatic antioxidants (such as superoxide dismutase (SOD), glutathione (GSH) and catalase (CAT)).

Vitamin E (VE), tocopherol, is a common antioxidant, which is easy to obtain and elicits few side effects on cell metabolism. Vitamin E is a chain-breaking antioxidant and the main fat soluble antioxidant in cells. At the cellular level, vitamin E provides its phenolic hydrogen to lipid peroxy radicals and converts them into less destructive substances, e.g. α-Tocopherol free radical [19]. As such, VE can help block the free radical chain reaction in part by maintaining the integrity of long-chain polyunsaturated fatty acids in the membrane, which is associated with the biological activity and signal transduction events of these fatty acids [18].VE can reduce damage in various organs by reducing oxidative stress and helping alleviate transport pressure [20–23]. In addition, vitamin E is considered as an effective method to reduce the oxidation of meat [24].

Yaks offer daily requirements such as meat, milk, hair, skin, fodder, and fuel to Tibetan herders. The forage quality and quantity in Tibet is poor in the winter, and yaks’ energy and protein intake is lower than what is required for survival. The weight of yaks is lost by around 25% after a cold season, which posing a severe threat to the sustainable development of yak industry. The establishment of reproduction in the west and finishing in the south of yaks will solve the contradiction between the grass and livestock in the west pastoral zone, meanwhile the poor meat quality and low economic benefits of yaks production will be improved. As far as we know, this study is first to report the improvement effect of VE on transport stress in yaks. Our hypothesis was that VE could relieve the post transportation stress in yak. To address this hypothesis, VE was injected once upon the arrival of yaks to the destination farm followed by analysis of VE serum concentrations, biochemical indices, antioxidants and metabolomics profiles.

## Materials and methods

### Ethical approval

This study was approved by the ethics committee of the Chongqing Academy of animal sciences (Approval Number: xky‐20180716).

### Animal experiment and sample collection

Five yaks aged 4 years in good health and free of clinical disease were selected for this study. All yaks were transported for approximately 34 hours (2100 km) on a tarred road from Riwoqe county (Tibet Autonomous Region) at an altitude of 3900 meters to Rongchang District (Chongqing Municipality) at an altitude of 400 meters. The same five yaks were sampled before and after VE injection. Once the yaks arrived at the farm, blood samples were collected immediately via jugular venipuncture and these samples served as the baseline control (CON_VE). Then, 40 mg/kg [25] VE was injected intramuscularly and blood samples collected after 10 days before the morning feeding. These represented the VE treatment (VE) samples. Serum extraction was carried out through centrifuging at 3000 rpm for 10 min at 4°C. Serum samples were stored at -80°C immediately for biochemical index determination, antioxidant index determination and metabolomics analysis. All animals were fed the same diet mainly including hay, fresh pennisetum hydridum, cornmeal and soybean meal twice daily at 8:30 AM and 5:00 PM, and fresh water was offered at all times during the 10 days.

### Blood biochemical index determination

Blood biochemical analysis was performed with an automatic biochemical analyzer (Beckman Coulter AU680). Briefly, all items were measured using colorimetric assays (modified kinetic Jaffe method), turbidimetry, latex agglutination, homogeneous EIA and indirect ISE according to the Beckman Coulter AU680 analyzer specifications.

### Antioxidant index determination

The level or activity of VE, lipid peroxide (LPO), malondialdehyde (MDA), reactive active oxygen species (ROS), total antioxidant capacity (T-AOC), superoxide dismutase (SOD), peroxidase (POD), catalase (CAT), thioredoxin peroxidase (TPX) glutathione reductase (GR) glutathione s-transferase (GSH-ST), glutathione peroxidase (GSH-PX) and glutathione (GSH) in blood serum were measured using commercial assay kits (Nanjing Jiancheng Bioengineering Institute, Jiangsu, China) according to the manufacturer’s protocols.

### Metabolite extraction

A total of 20 μL of sample was transferred to an EP tube. After adding 80 μL of extract solution (acetonitrile: methanol = 1:1, containing isotopically-labelled internal standard mixture), the samples were vortexed for 30 seconds, sonicated for 10 min in an ice-water bath, and incubated at -40°C for 1 hour to precipitate proteins. The sample was then centrifuged at 4°C for 15 minutes at 12000 rpm (RCF = 13800 (g), R = 8.6 cm). The supernatant that resulted was transferred to a new glass vial for analysis. The quality control (QC) sample was made by combining an equal aliquot of supernatant from each sample.

### UHPLC-MS-MS analysis

LC-MS/MS analyses were performed using an UHPLC system (Vanquish, Thermo Fisher Scientific) with a UPLC BEH Amide column (2.1 mm × 100 mm, 1.7 μm) coupled to a Q Exactive HFX mass spectrometer (Orbitrap MS, Thermo). The mobile phase consisted of 25 mmol/L ammonium acetate and 25 ammonia hydroxide in water (pH = 9.75). The temperature of the auto-sampler was 4°C, and the injection volume was 2 μL. The QE HFX mass spectrometer was chosen for its capacity to obtain MS/MS spectra using the acquisition software’s information-dependent acquisition (IDA) mode (Xcalibur, Thermo). The acquisition program constantly assessed the complete scan MS spectrum in this mode. The following ESI source conditions were used: 30 Arb sheath gas flow rate, 25 Arb aux gas flow rate, 350°C capillary temperature, complete MS resolution 60000, MS/MS resolution 7500, collision energy 10/30/60 in NCE mode, and spray voltage 3.6 kV (positive) or—3.2 kV (negative).

### Data analysis

First, if metabolite features were detected in < 20% of experimental samples or in < 50% of QC samples they were removed from data analysis. Then the missing values of raw data were filled up by half of the minimum value. In addition, an internal standard normalization method was employed in this data analysis. Lastly, features with RSD > 30% were removed from the subsequent analysis. The resulting three-dimensional data involving the peak number, sample name, and normalized peak area were fed to R package metaX for principal component analysis (PCA) and orthogonal projections to latent structures-discriminate analysis (OPLS-DA). To further prove the reliability of the model, the permutation order of classification variable Y was randomly changed by a permutation test, which established a value 200 times. A fold change analysis (FC analysis) and T-test of the data were used to detect and identify differential metabolites between the VE and the baseline. A student’s t-test *P* < 0.05 and variable importance in the projection (VIP) > 1 were used to detect significant changes. Metabolites were visualized by volcano plots using the ggplot2 package of R software. In addition, commercial databases including KEGG (http://www.kegg.jp) and MetaboAnalyst (http://www.metaboanalyst.ca/) was utilized for metabolic pathway analysis.

Data on blood biochemical indices and blood antioxidant indices were analyzed using a MIXED model in SAS (version 9.3; SAS Institute Inc, Cary, NC, USA) with VE injection as a fixed effect and animal (yak) as the random effect. Treatment means were obtained via the LSMEANS option and separated using the PDIFF option with significance at *P*<0.05. Data are presented as means ± standard error.

## Results

### Vitamin E concentration in blood

**Fig 1:**
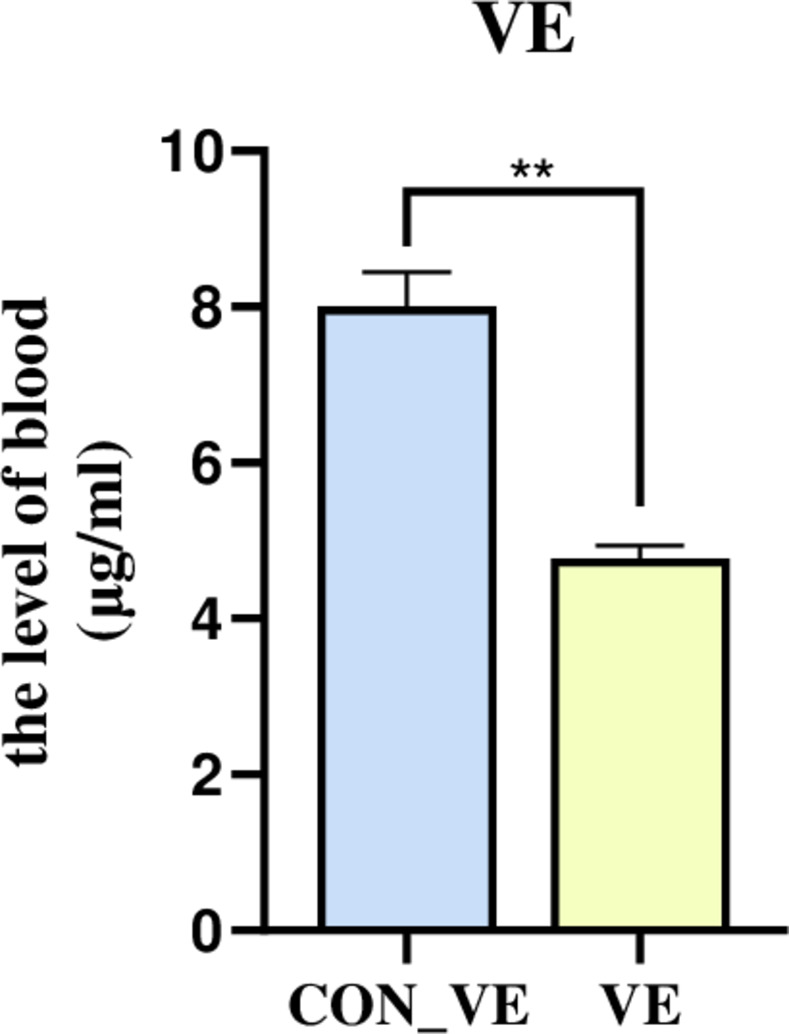
Blood serum VE concentration before and after VE injection after transportation in yak.

The VE content after injection of VE was significantly lower than the baseline (CON_VE) (Fig 1).

### Blood biochemical parameters

A total of 24 biochemical indices were detected in this study (Table 1). Compared with CON_VE, VE injection significantly (*P*<0.05) decreased concentrations of ALT, AST, ALP, ALBⅡ, DBIL, IBIL, UREA, TG, HDLC, GLU, A/G, TBIL and TC. However, VE injection led to greater (*P*<0.05) AST/ALT, TBA, CHE, CREA-S and LAC relative to CON_VE. There was no difference (*P*>0.05) in GGT, TP, GLO, LDLC and LDH observed between CON_VE and VE.

**Table 1:** Blood biochemical indices before and after VE injection after transportation in yak.

Items	Treatment^1^		SEM	*P*-Value
	CON_VE	VE		
ALT(U/L)	44.00^a^	20.80^b^	2.44	< 0.01
AST(U/L)	111.1^a^	71.67^b^	9.45	0.01
GGT(U/L)	9.20	10.60	0.76	0.31
ALP(U/L)	108.83^a^	94.17^b^	5.08	< 0.01
AST/ALT	2.49^b^	3.33^a^	0.15	< 0.01
TBA	12.52^b^	34.98^a^	4.25	< 0.01
TP(g/L)	65.35	63.10	1.23	0.32
ALBⅡ(g/L)	39.87^a^	37.30^b^	0.65	0.01
GLO(g/L)	25.48	24.98	1.33	0.08
A/G	1.60^b^	1.43^a^	0.08	0.05
T-Bil(μmol/L)	13.08^b^	5.46^a^	1.77	0.02
D-Bil(μmol/L)	4.53^a^	1.33^b^	0.59	0.01
IDBIL(μmol/L)	8.55^a^	3.92^b^	0.98	0.01
CHE(U/L)	111.67^b^	116.67^a^	4.84	< 0.01
UREA(mmol/L)	8.77^a^	6.18^b^	0.44	< 0.01
CREA-S(μmol/L)	149.12^b^	162.48^a^	5.62	< 0.01
TC(mmol/L)	2.33^a^	1.98^b^	0.098	0.03
TG(mmol/L)	0.21^a^	0.14^b^	0.03	< 0.01
HDL-C(mmol/L)	1.51^a^	1.27^b^	0.04	< 0.01
LDL-C(mmol/L)	0.73	0.70	0.07	0.43
GLU(mmol/L)	3.16^a^	2.48^b^	0.14	0.01
l-lactate detection (mmol/L)	3.05^b^	4.69^a^	0.33	0.01
LDH(U/L)	969.00^a^	790.75^b^	52.00	0.07

^1^(VE, Injection of VE groups; CON_VE, VE control group)

^a, b^ Letters means within a row that do not have a common superscript letter differ, *P*< 0.05. (ALT, glutamate pyruvic transaminase; AST, aspartate aminotransferase; GGT, glutamyl transferase; ALP, alkaline phosphatase; TBA, Total bile acid; TP, Total protein; ALBⅡ, albumin; GLO, Globulin; T-Bil, total bilirubin; D-Bil, direct bilirubin; IDBIL, indirect bilirubin; CHE, cholinesterase; CREA-S, creatinine; TC, total cholesterol; TG, triglyceride; HDL-C, high density lipoprotein; LDL-C, low density lipoprotein; GLU, glucose; LDH, lactate dehydrogenase)

### Evaluation of oxidative stress

**Fig 2:**
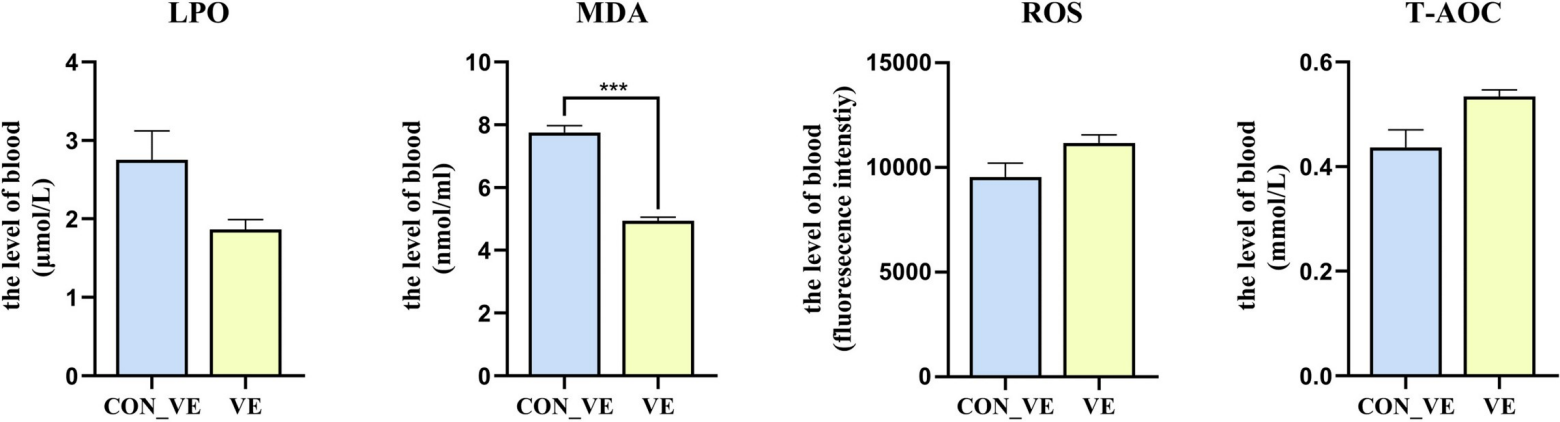
The blood serum level of LPO, MDA, ROS and T-AOC before and after VE injection after transportation in yak.

The effect of VE injection on serum level of LPO, MDA, ROS and T-AOC is shown in Fig 2. Compared with the control group, VE significantly reduced the blood level of MDA. There were tendencies for increases in the serum level of ROS (*P* = 0.053), while LOP (*P* = 0.087) tended to decrease. And an increasing of T-AOC with *P* = 0.101 was also observed with VE injection.

**Fig 3:**
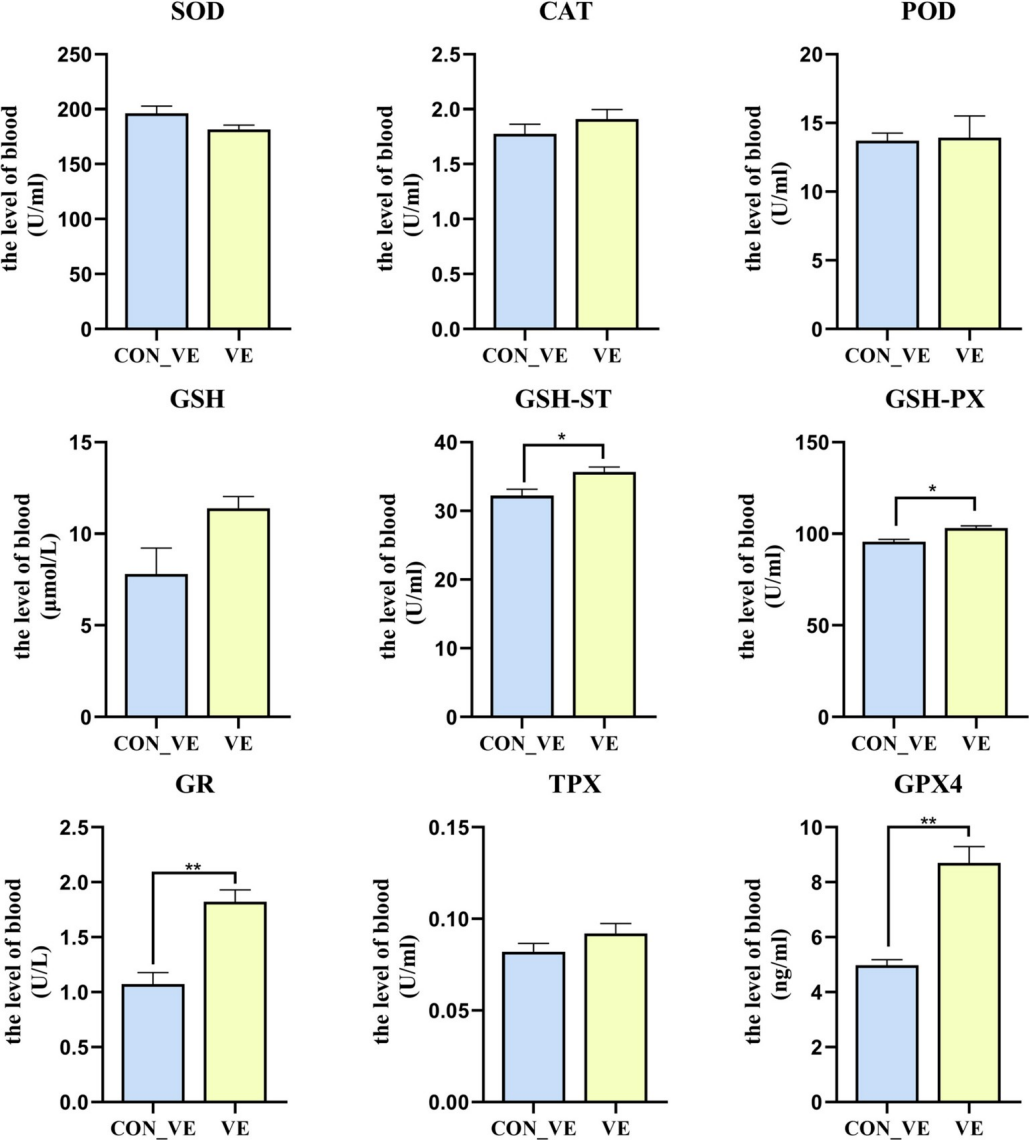
The blood serum level of SOD, CAT, POD, GSH, GSH-ST, GSH-PX, GR, TPX and GPX4 before and after VE injection after transportation in yak.

The effect of VE injection on the blood serum SOD, CAT, POD, GSH, GSH-ST, GSH-PX, GR, GPX4 and TPX level of yak is shown in Fig 3. Compared with CON_VE, VE injection led to greater (*P*<0.05) serum GSH-ST, GSH-PX, GR and GPX4. No difference (*P*>0.05) was detected for SOD, CAT, POD, GSH and TPX between CON_VE and VE.

### Metabolomics analysis of serum

**Fig 4:**
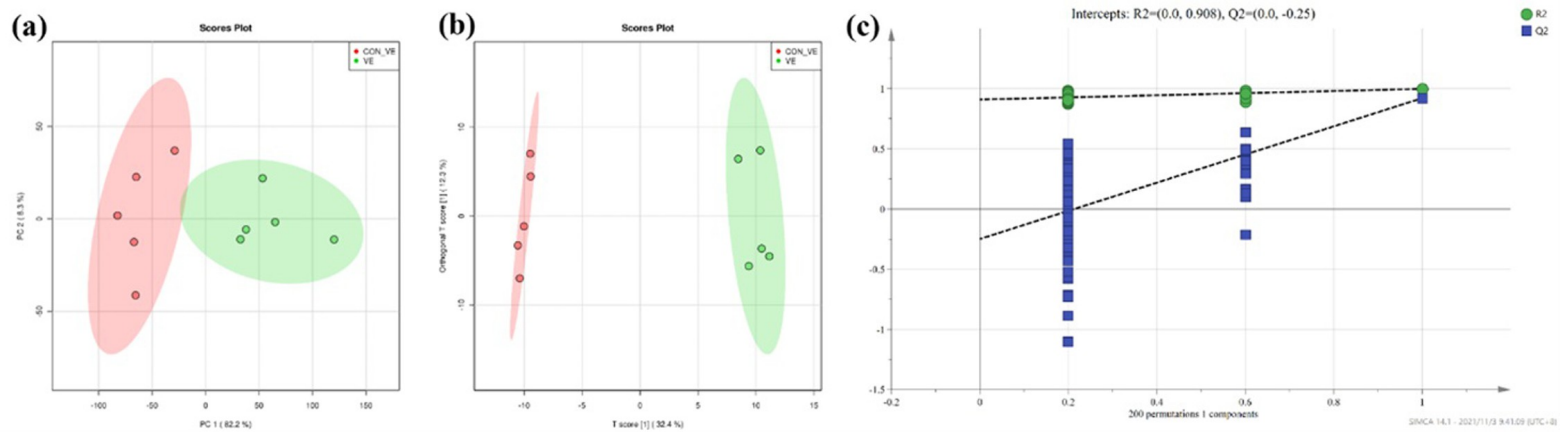
Metabolomics of PCA analysis with serum sample before and after VE injection after transportation in yak. (a), OPLS-DA analysis (b) and permutation test of OPLA-DA (c). CON_VE = blood sample was collected once upon arriving the farm before injection.VE = blood sample was collected after injection on the 10^th^ day. PCA = principal component analysis, the red represent CON_VE, and the green represent VE injection. OPLS-DA = orthogonal partial least squares discriminant analysis, the red represent CON_VE, and the green represent VE injection.

A total of 343 compounds were identified and quantified based on the LC-MS/MS analysis. The multivariate analysis of PCA and OPLS-DA revealed separate clusters between the CON_VE group and VE group (Fig 4A and 4B). The R2Y and Q2 parameters are both used to evaluate the reliability and predictive ability of the model in the OPLS-DA analysis. The R2Y was greater than 0.908 suggesting good reliability of the model used in this study. The Q2 was greater than 0.75 also suggesting good predictive ability of the model used. The 200 permutation tests performed avoided overfitting of the OPLS-DA model. Both pR2Y and pQ2 were less than 1.0, which further indicated good robustness and validity of the model (Fig 4C).

**Fig 5:**
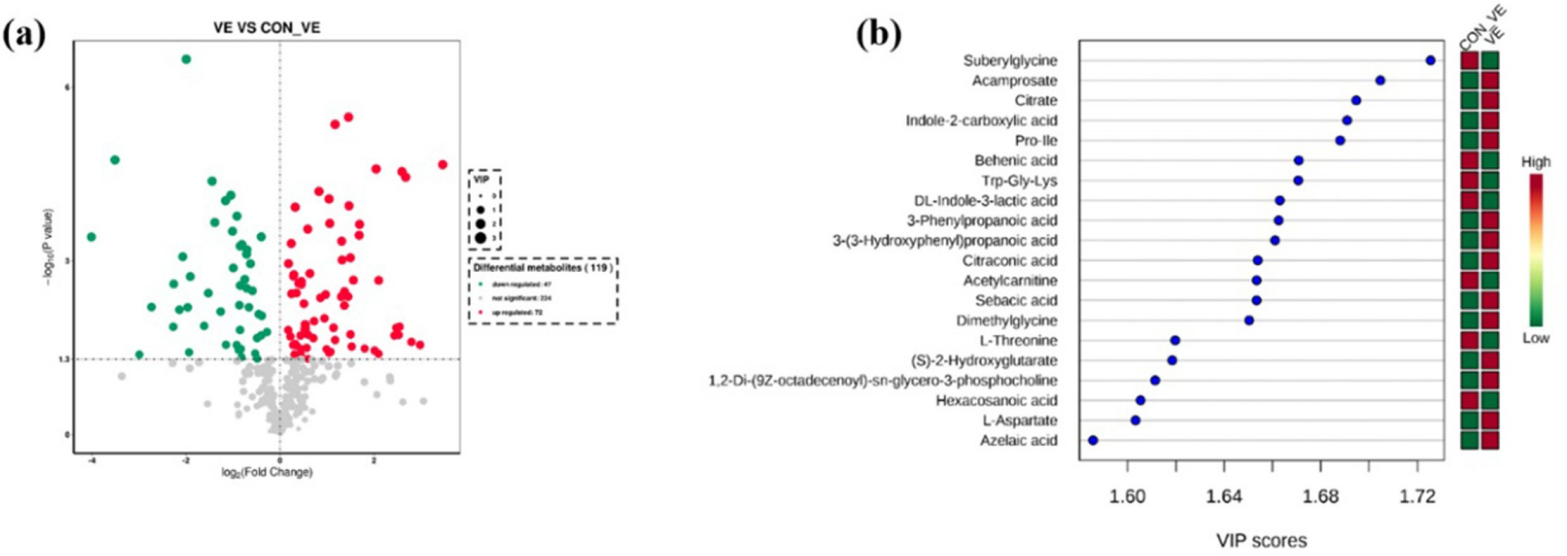
Differential metabolites in serum samples before and after VE injection in yak. (a) Volcano plots of the difference metabolites. Each point in the volcanic map represents a metabolite, red and green dots indicate up-regulated and down-regulated metabolites respectively. Metabolites with no difference are shown in gray. (b) Metabolites are ranked by variable importance in projection analysis (VIP) of respective groups. The top 20 important metabolites were arranged from top to bottom according to intracellular concentration. The red box represents a high concentration of the molecule and the green box represents low concentration.

The effect of VE injection on metabolomics profiles of yak serum are shown in Fig 5. In total we identified 119 differentially altered metabolites (*P*<0.05 and VIP>1.0) as a result of VE injection (Fig 5A). Of those, 72 metabolites increased and 47 decreased (Fig 5A). More details about differential metabolites are reported in S1 Table. The top 20 differentially altered metabolites are depicted in VIP plots (Fig 5B). Among those that increased in response to VE injection were Acamprosate, Citrate, Indole-2-carboxylic acid, Pro-Ile, 3-Phenylpropanoic acid, 3-(3-Hydroxyphenyl) propanoic acid, Citraconic acid, Sebacic acid, Dimethylglycine, (S)-2-Hydroxyglutarate, 1,2-Di-(9Z-octadecenoyl)-sn-glycero-3-phosphocholine, L-Aspartate and Azelaic acid. Compared with CON_VE, concentrations of Suberylglycine, Behenic acid, Trp-Gly-Lys, DL-Indole-3-lactic acid, Acetylcarnitine, L-Threonine and Hexacosanoic acid decreased after VE injection.

**Fig 6:**
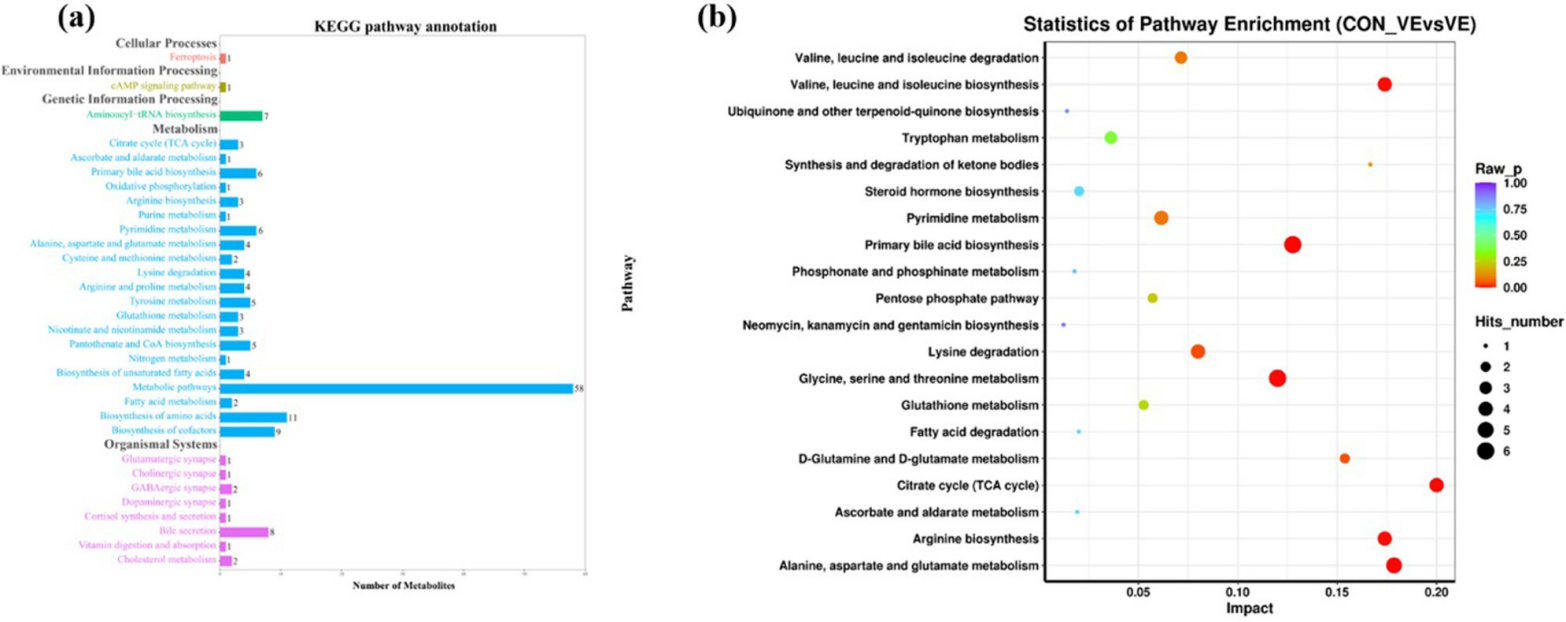
KEGG pathway annotation and enrichment analysis of metabolites in yak serum before and after VE injection. (a) The KEGG pathway annotation analysis of metabolism pathway. The horizontal axis is the number of differential metabolites, and the vertical axis is the pathway. Different colors represent different secondary-level of pathways classification in the system. (b) The enrichment analysis of top 20 metabolism pathways. The color and size of each circle is based on *P*-values and pathway impact values respectively.

**Fig 7:**
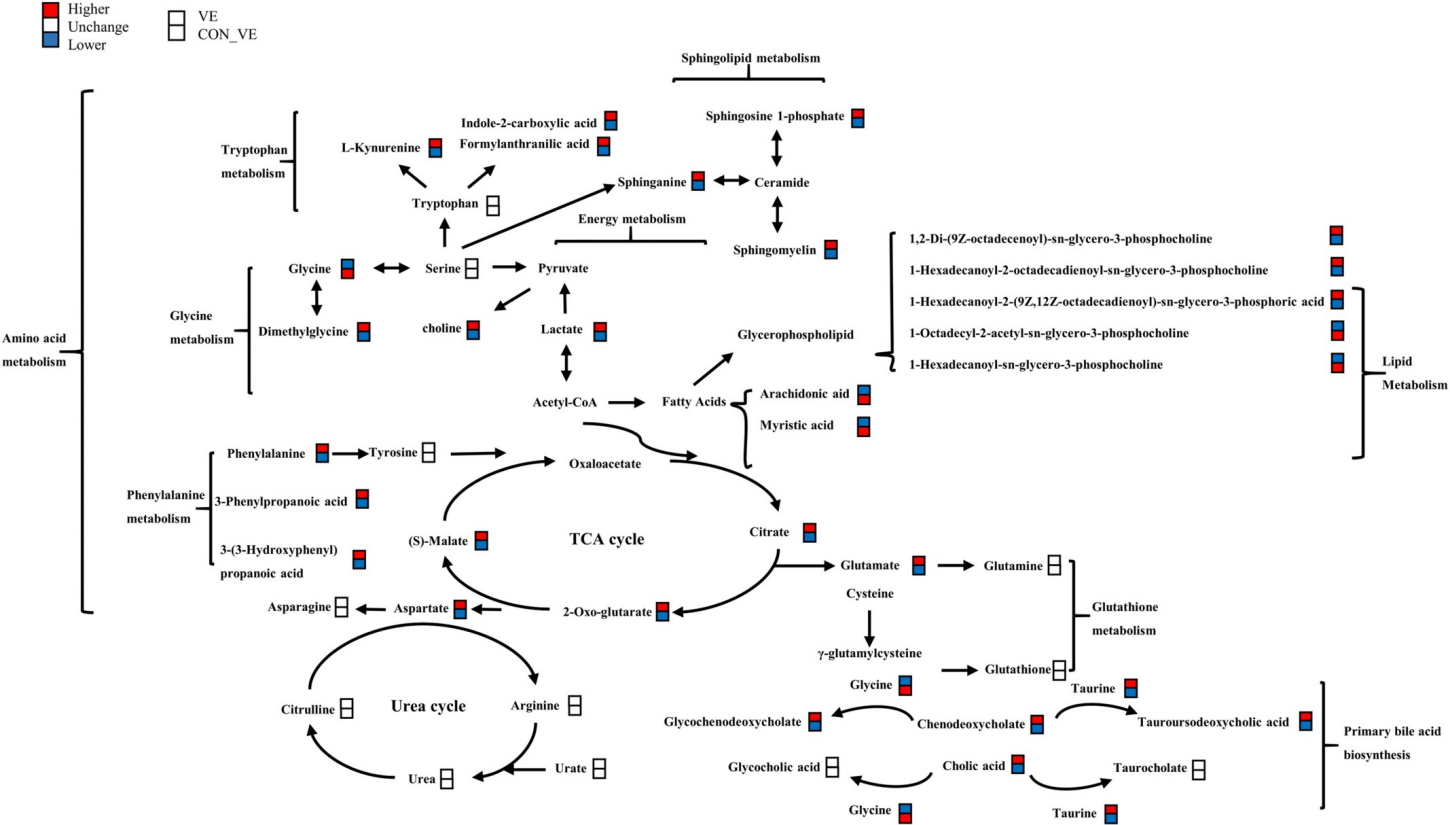
Metabolic network in yak serum induced before and after VE injection. The red boxes represents the higher concentration of metabolites and the blue boxes represents the low.

KEGG pathway annotation analysis was performed with the significantly (*P*<0.05, VIP> 1.0) altered metabolites for further biological function analysis (Fig 6A). Enriched pathways with *P*<0.05 are shown in Fig 6B. VE significantly affected metabolic pathways including citrate cycle, valine, leucine and isoleucine biosynthesis, arginine biosynthesis and alanine, aspartate and glutamate metabolism. Lastly, a metabolic network (Fig 7) was created to illustrate the interactions among differentially altered metabolites.

## Discussion

Transportation stress is a huge challenge to the health and production performance of yaks. To our knowledge, there are few reports dealing with the alleviation of transportation stress in yaks. Previous study have already demonstrated that the serum level of VE could not recover without VE supplementation until 26th day after transportation in beef steers [26]. Moreover, the liner relationship relative to serum level of VE was observed with before and after VE supplementation. Thus, the present work aimed to study the effects of before and after vitamin E injection on the health, antioxidant stress system and blood metabolism in yaks once arrived the destination farm after long-distance transportation.

### VE concentration

In this study, we found that compared with the control group, the vitamin E blood level in the VE group was lower, probably because of the increased demand for VE after transportation stress to drive antioxidant stress, and our injection volume is in short supply. Therefore, it is recommended to inject a higher dose of VE, or inject it continuously for several days. According to the description of Deters et al. [26], supplementing VE with the recommended amount (500 IU/steer daily) can improve the activity of the antioxidant enzyme SOD. Therefore, the amount of VE injection should consider the physiological needs of animals (such as growth, immune function, antioxidant defense).

### Biochemical indexes

The overall decrease in most of the liver function indices after VE indicated that it may have offered greater protection during oxidative stress. I-Bil, D-Bil [27, 28], T-Bil, ALP, AST and ALT are common indicators of liver disease. This response confirmed that increased supply of vitamin E during a stressful period can reduce hepatocyte injury [29]. Dey et al. [30] found that VE can reduce the increase in liver lipid peroxidation and limit injury mediated by oxidative stress.

Urea and creatinine are nitrogen-containing end products of metabolism and are clinically screening indicators of renal function. Urea is the main metabolite resulting from dietary protein and tissue protein conversion, and creatinine is a product of muscle creatine catabolism [31]. Dalmau et al. [32] evaluated the impact of transportation time in lambs and found that 24 hours after transportation the blood level of urea increased and the blood level of CREA decreased significantly. In our study, compared with the baseline, the blood level of urea decreased significantly and CREA increased significantly after VE injection, which suggested that VE injection helped yaks maintain proper kidney function.

### Oxidative stress markers

Several products of oxidative stress were determined. Among them, MDA decreased significantly with VE injection. MDA is the last product of lipid peroxidation [33, 34]. Plasma MDA concentration has been widely used in biology and medicine as the most common biomarker to evaluate lipid peroxidation [35]. Serum MDA level is positively correlated with the intensity of oxidative stress [36]. Tanko et al. found that a significant increase in plasma MDA concentrations was observed immediately after transportation [14]. Similarly, Surai et al. [37] found that increased vitamin E supplementation in the diet of breeder or cockerels increased antioxidant defenses and decreased lipid peroxidation. Thus, we speculated that transport stress increases the concentration of MDA in serum and vitamin E reduces it by antagonizing lipid peroxidation and cell damage, thereby contributing to alleviating oxidative stress.

### Antioxidant enzymes

We analyzed the chemicals to furtherly assess how the VE injection affected the antioxidant capacity in post-transit yaks. Following VE injection, an increasing in the serum levels of glutathione peroxidase, glutathione reductase, and glutathione S-transferase was observed, which are with the ability to scavenge peroxides. Glutathione reductase performs an auxiliary function in the antioxidant mechanism associated with glutathione. Reduction of oxidized glutathione restores its antioxidant properties, thereby enabling its participation in reactions catalyzed by glutathione peroxidase and glutathione S-transferase leading to elimination of ROS [14, 38]. In the present study, supplementation with VE reduced the production of lipid peroxidation products and increased the serum level of GSH-ST, GSH-PX, GR and GPX4 in the serum. This is similar to the research of Cahide et al. [39], that vitamin E supplementation caused activation of GSH related enzymes. GSH-PX is the first line of cellular defense in the body, and GSH-ST was confirmed as the secondary antioxidant enzymes in the antioxidant defense system GSH-ST [40]. Both of these two antioxidant enzymes are expected as being capable of coping with oxidative stress in transport stress. Similarly, Min et al. [41]. found that birds supplemented with VE improved antioxidant capability and immune function through up-regulating the relative expression of GSH-PX mRNA under oxidative stress. In our study, GSH-PX and GSH-ST of yaks increased significantly after VE injection, indicating that the body’s ability to cope with increased oxidative stress was enhanced. Together, these data suggested that VE improves the oxidative stress status of yaks after transportation stress by increasing the serum level of GSH related oxidoreductase.

### Metabolomics studies

To further understand the regulatory mechanisms associated with VE supply in yaks after transportation, blood metabolomics was performed.

#### Lipid metabolism

The body produces oxygen free radicals through enzymatic and non-enzymatic systems, which can attack polyunsaturated fatty acids in the membrane and trigger lipid peroxidation. Lipids undergo a series of complex peroxidation reactions, leading to the catabolism of polyunsaturated fatty acids and the formation of highly active unsaturated aldehydes. The end products of lipid peroxidation are reactive aldehydes, such as arachidonic acid. In our study, we found that serum levels of NEFA (oleic acid) and saturated fatty acids (tridecanoic acid, myristic acid and behenic acid) were significantly lower in the VE injection groups. Pascual Alonso et al. found that NEFA increased immediately after transportation [9], suggesting body fat mobilization to generate energy [42]. The lower concentration of arachidonic acid after VE injection indicated that it helped reduce the content of arachidonic acid. Arachidonic acid is an important stress metabolic marker, which induces inflammation [43] and abnormal lipid metabolism [44–46]. This is similar to the results of Obajimi et al. [47], where tocopherol significantly decreased the release of arachidonic acid stimulated by oxidants, while ascorbic acid significantly increased the release. Peeters et al. [22] also found that exogenous VE in the feed reduced NEFA after simulation of transport state through vibration. Overall, these results suggested that VE can reduce the production of lipid peroxidation to alleviate oxidative stress.

In the present work, a significant change of lysophosphatidylethanolamine (LysoPE), lysophosphatidylcholine (LysoPC), phosphocholine and choline indicated that the presence of VE could alleviate road transportation stress partly through regulating the glycerophospholipid biosynthesis pathway. Glycerophospholipids are the main components of cell membranes and have a wide range of biological functions in cell proliferation, differentiation, and apoptosis [48, 49]. LysoPCs are regarded as the products of fatty acid oxidation [50, 51], when all phospholipids are oxidized, pore formation can occur. This will allow reactive species, such as reactive oxygen and nitrogen species, to enter the cell and cause oxidative damage to intracellular macromolecules such as DNA or proteins [52]. Specific, the LysoPC (16:0/0:0) decreased significantly after VE injection demonstrating that it helped alleviate fatty acid oxidation and facilitate cell membrane biosynthesis. Based on these results, it seem plausible that VE is likely to protect the cell membrane integrity of the animals by protecting against lipid peroxidation under stressful conditions, which we will further investigate in subsequent experiments.

In addition, we found the blood concentrations of sphinganine, sphingosine 1-phosphate and sphingomyelin increased significantly after VE injection. Sphingolipids and its products are closely related to cell function and status [53]. This result further demonstrates that VE may be able to reduce oxidative stress-induced damage by protecting cell membranes.

#### Energy metabolism

Carbohydrate metabolism refers to a series of complex chemical reactions among which those of glucose (Glu) and glycogen (GN) are the most important from the energy standpoint. In terms of aerobic metabolism, malate, citrate and α-Oxo-glutarate (OGC) are important metabolites in the tricarboxylic acid cycle, and citrate is the starting point of the cycle. Takemoto et al. reported that in cattle citrate decreased immediately after transport [54]. There was a marked decrease in plasma malate, citrate and α-Oxo-glutarate in plasma metabolome, and Glu in blood biochemical results after VE injection, indicating that TCA cycle might have been stimulated in order to help meet cellular energy demands after transportation. Citrate exhibits anti-oxidant and anti-inflammatory properties in different cells and tissues [55], and in one study citric acid reduced transport stress in cattle [54]. The increase in citrate may also suggest enhanced antioxidant and anti-inflammatory capacity in the yaks.

#### Amino acid metabolism

In this study, the levels of the phenylalanine and its metabolites (3-Phenylpropanoic acid and 3-(3-Hydroxyphenyl) propanoic acid (3HPPA)) in the serum of the yaks decreased after VE administration. It is possible that HPPA helped eliminate the free radicals produced by red blood cells, thus, contributing to alleviating oxidative stress [56]. These changes demonstrate that VE administration might alleviate oxidative by promoting phenylalanine metabolism.

#### Cortisol

Circulating cortisol is the most predominant measure of stress studied in cattle [17]. The fact that plasma urea and cortisol decreased markedly after VE suggested an increase in protein and nucleic acid metabolism in muscle due to an increase in cortisol concentration during transportation [57]. The increase of cortisol concentration in blood is often an indicator of animal psychological [1] and transportation stress [10, 20, 58, 59]. Circulating cortisol is the most predominant measure of stress studied in cattle [17]. In our study, cortisol levels decreased significantly ten days after VE injection. The results showed that VE could reduced the concentration of cortisol and alleviated oxidative stress, which is consistent with the conclusions of previous studies. In the study of Sathya et al. [60], vitamin E supplementation reduced plasma cortisol in dystocic buffalo. Similar to our results, Peeters et al. [22]. showed that VE supplementation in pigs produced the least cortisol during stressful periods.

Taken together, these metabolomics data indicated that VE supplementation stimulated a protective effect on the oxidative damage induced by transportation. VE can promote the tricarboxylic acid cycle to help meet the increased energy demands after transportation stress, and promote amino acid metabolism to synthesize antioxidants to form an antioxidant defense system. Overall, our study indicated that VE could protect cell membrane structure by reducing lipid peroxidation as a way to alleviate oxidative stress after transport stress in yaks. These findings underscored the significance of assessing oxidant status in transported yaks to help ameliorate any stress. In that context, supplementation of VE seems effective as supportive treatment geared towards enhancing productivity and profitability of yak farming.

## Conclusions

Because of the production systems involving yak, the need for long haul transportation of these animals leads to serious stress, which results in huge economic losses due to poor health and substandard meat quality. Supplemental VE alleviates transport stress through improving the antioxidant ability of the animal, protecting the liver and kidney, reducing lipid peroxidation and maintaining cell membrane structure. Considering the decrease in serum VE level we detected after VE injection, future studies should pay more attention on the dose of VE injection in yak after transportation.

## Supporting information

S1 Table The concentration of metabolites in the CON_VE&VE groups. (DOCX)

S2 Table The concentration of metabolites in the metabolites relevant pathway .network. (DOCX)
