# Supplementary material for: Vitamin E supplementation improves post-transportation systemic antioxidant capacity in yak
Source: PLoS One. 2022 Dec 2;17(12):e0278660. doi: 10.1371/journal.pone.0278660 (PMC9718397; doi:10.1371/journal.pone.0278660)
Supplement: S1 Table — (DOCX) [file pone.0278660.s001.docx]

**Supplementary Information**

**Supplemental Table 1 The concentration of metabolites in the CON_VE&VE groups**

| Metabolites | VIP | *P*-Value | UP/DOWN |
| --- | --- | --- | --- |
| Suberylglycine | 1.7255 | <0.0001 | DOWN |
| Acamprosate | 1.7047 | <0.0001 | UP |
| Citrate | 1.6947 | <0.0001 | UP |
| Indole-2-carboxylic acid | 1.6909 | <0.0001 | UP |
| Pro-Ile | 1.6880 | <0.0001 | UP |
| Behenic acid | 1.6708 | <0.0001 | DOWN |
| Trp-Gly-Lys | 1.6707 | <0.0001 | DOWN |
| DL-Indole-3-lactic acid | 1.6630 | 0.0001 | DOWN |
| 3-Phenylpropanoic acid | 1.6626 | <0.0001 | UP |
| 3-(3-Hydroxyphenyl)propanoic acid | 1.6611 | <0.0001 | UP |
| Citraconic acid | 1.6539 | 0.0001 | UP |
| Sebacic acid | 1.6534 | 0.0001 | UP |
| Acetylcarnitine | 1.6534 | 0.0001 | DOWN |
| Dimethylglycine | 1.6503 | 0.0001 | UP |
| L-Threonine | 1.6197 | 0.0002 | DOWN |
| (S)-2-Hydroxyglutarate | 1.6185 | 0.0002 | UP |
| "1,2-Di-(9Z-octadecenoyl)-sn-glycero-3-phosphocholine" | 1.6114 | 0.0001 | UP |
| Hexacosanoic acid | 1.6054 | 0.0002 | DOWN |
| L-Aspartate | 1.6033 | 0.0003 | UP |
| L-Palmitoylcarnitine | 1.6012 | 0.0003 | DOWN |
| Azelaic acid | 1.5857 | 0.0002 | UP |
| Succinate | 1.5855 | 0.0008 | DOWN |
| Glycine | 1.5797 | 0.0004 | DOWN |
| Arachidic acid | 1.5734 | 0.0004 | DOWN |
| N4-Acetylcytidine | 1.5683 | 0.0004 | UP |
| L-Malic acid | 1.5513 | 0.0005 | UP |
| "Benzeneethanol, 2-hydroxy-" | 1.5445 | 0.0005 | UP |
| 3-Methylhistamine | 1.5425 | 0.0006 | DOWN |
| Oleic acid / trans-Vaccenic acid | 1.5418 | 0.0006 | DOWN |
| Alpha-ketoisovaleric acid | 1.5406 | 0.0011 | DOWN |
| Hippuric acid | 1.5379 | 0.0009 | UP |
| 1-Octadecyl-2-acetyl-sn-glycero-3-phosphocholine | 1.5362 | 0.0008 | DOWN |
| D-Glucosaminic acid | 1.5320 | 0.0021 | DOWN |
| alpha-Linolenic acid | 1.5245 | 0.0005 | DOWN |
| trans-cinnamate | 1.5210 | 0.0011 | UP |
| Alpha-N-Phenylacetyl-L-glutamine | 1.5201 | 0.0010 | UP |
| L-Glutamate | 1.5163 | 0.0023 | UP |
| "3,4-Dihydroxybenzoate (Protocatechuic acid)" | 1.5128 | 0.0022 | UP |
| Stearoylcarnitine | 1.5106 | 0.0019 | DOWN |
| DL-lactate | 1.5086 | 0.0017 | UP |
| S-Methyl-L-thiocitrulline | 1.5058 | 0.0018 | UP |
| Gly-Glu | 1.5034 | 0.0013 | DOWN |
| Tyramine | 1.4979 | 0.0017 | UP |
| Phe-Pro | 1.4969 | 0.0034 | UP |
| L-Kynurenine | 1.4781 | 0.0026 | UP |
| Tridecanoic acid (Tridecylic acid) | 1.4726 | 0.0027 | DOWN |
| Estrone-3-glucuronide | 1.4698 | 0.0025 | DOWN |
| Choline | 1.4686 | 0.0037 | UP |
| Cytidine | 1.4634 | 0.0025 | UP |
| alpha-ketoglutarate | 1.4627 | 0.0042 | UP |
| 4-Hydroxy-3-methylbenzoic acid | 1.4621 | 0.0042 | UP |
| Hydrocortisone | 1.4602 | 0.0036 | DOWN |
| Nervonic acid | 1.4586 | 0.0033 | DOWN |
| "3,4-Dihydroxyphenylacetic acid" | 1.4579 | 0.0022 | UP |
| D-Quinovose | 1.4536 | 0.0030 | DOWN |
| "1,4-Dihydroxybenzene" | 1.4392 | 0.0059 | UP |
| Formylanthranilic acid | 1.4197 | 0.0038 | UP |
| L-Phenylalanine | 1.4193 | 0.0036 | UP |
| Phe-Trp | 1.4066 | 0.0070 | DOWN |
| L-Valine | 1.4017 | 0.0089 | DOWN |
| N6-(1-Iminoethyl)-L-lysine | 1.3994 | 0.0064 | DOWN |
| N-Acetyl-L-glutamate | 1.3871 | 0.0083 | DOWN |
| 2-Hydroxyphenylacetic acid | 1.3784 | 0.0055 | UP |
| 1-Hexadecanoyl-2-octadecadienoyl-sn-glycero-3-phosphocholine | 1.3777 | 0.0044 | UP |
| Leu-Lys-Arg | 1.3754 | 0.0059 | DOWN |
| Myristic acid | 1.3709 | 0.0075 | DOWN |
| "15-Deoxy-delta-12,14-PGJ2" | 1.3697 | 0.0063 | DOWN |
| cis-9-Palmitoleic acid | 1.3476 | 0.0063 | DOWN |
| Gly-Arg | 1.3314 | 0.0143 | UP |
| Met-Cys-Arg | 1.3258 | 0.0157 | DOWN |
| N5-(1-Iminoethyl)-L-ornithine | 1.3256 | 0.0133 | DOWN |
| Adipic acid | 1.3227 | 0.0126 | UP |
| Biliverdin | 1.3135 | 0.0138 | DOWN |
| Val-His | 1.3118 | 0.0110 | UP |
| 6-Phospho-D-gluconate | 1.3098 | 0.0148 | UP |
| Lys-Gln-Arg | 1.3053 | 0.0187 | UP |
| Phenylpyruvate | 1.2966 | 0.0098 | UP |
| Acetoacetic acid | 1.2956 | 0.0188 | UP |
| Creatine | 1.2947 | 0.0194 | DOWN |
| 18.beta.-Glycyrrhetinic acid | 1.2866 | 0.0216 | DOWN |
| Pseudouridine | 1.2679 | 0.0151 | UP |
| 4-Guanidinobutyric acid | 1.2637 | 0.0195 | UP |
| Malonic acid | 1.2594 | 0.0303 | UP |
| Glycochenodeoxycholate | 1.2562 | 0.0138 | UP |
| Coniferyl aldehyde | 1.2514 | 0.0203 | UP |
| Pantothenate | 1.2506 | 0.0126 | UP |
| Taurodeoxycholic acid | 1.2500 | 0.0144 | UP |
| Leu-Gln-Arg | 1.2486 | 0.0252 | UP |
| Arachidonic Acid (peroxide free) | 1.2478 | 0.0170 | DOWN |
| 6-Benzylaminopurine | 1.2424 | 0.0233 | UP |
| "1-Hexadecanoyl-2-(9Z,12Z-octadecadienoyl)-sn-glycero-3-phosphoric acid" | 1.2339 | 0.0217 | UP |
| Vigabatrin | 1.2332 | 0.0158 | UP |
| Chenodeoxycholate | 1.2297 | 0.0355 | UP |
| N-Palmitoyltaurine | 1.2277 | 0.0346 | DOWN |
| Trimethylamine N-oxide | 1.2259 | 0.0284 | UP |
| 1-Phenoxy-2-propanol | 1.2242 | 0.0282 | DOWN |
| L-Histidine | 1.2235 | 0.0271 | UP |
| Glycodeoxycholic acid | 1.2161 | 0.0195 | UP |
| 1-Hexadecanoyl-sn-glycero-3-phosphocholine | 1.2082 | 0.0285 | DOWN |
| Tauroursodeoxycholic acid | 1.2056 | 0.0190 | UP |
| (S)-2-aminobutyric acid | 1.2053 | 0.0416 | UP |
| 16-Hydroxypalmitic acid | 1.2008 | 0.0333 | DOWN |
| Cholic acid | 1.1992 | 0.0403 | UP |
| Taurine | 1.1848 | 0.0319 | UP |
| Pro-Ser | 1.1799 | 0.0388 | UP |
| Dehydroascorbic acid (Oxidized vitamin C) | 1.1775 | 0.0398 | DOWN |
| Indole | 1.1773 | 0.0461 | DOWN |
| 1-Methylnicotinamide | 1.1738 | 0.0380 | DOWN |
| Benzoic acid | 1.1683 | 0.0295 | UP |
| Bilirubin | 1.1488 | 0.0416 | DOWN |
| D-Proline | 1.1417 | 0.0285 | UP |
| Thymidine | 1.1368 | 0.0492 | DOWN |
| Sphingomyelin (d18:1/18:0) | 1.1286 | 0.0403 | UP |
| Cytosine | 1.1264 | 0.0331 | UP |
| D-erythro-Sphingosine-1-phosphate | 1.1225 | 0.0495 | UP |
| Glycolithocholic acid | 1.1221 | 0.0373 | UP |
| 3-Ureidopropionate | 1.1216 | 0.0444 | UP |
| Taurochenodeoxycholate | 1.1206 | 0.0328 | UP |
| Sphinganine | 1.0906 | 0.0336 | UP |
