# Supplementary material for: Vitamin E supplementation improves post-transportation systemic antioxidant capacity in yak
Source: PLoS One. 2022 Dec 2;17(12):e0278660. doi: 10.1371/journal.pone.0278660 (PMC9718397; doi:10.1371/journal.pone.0278660)
Supplement: S2 Table — (DOCX) [file pone.0278660.s002.docx]

**Supplementary Information**

**Supplemental Table 2 The concentration of metabolites in the metabolites relevant pathway network**

| Metabolites | VIP | *P*-Value | VE&CON_VE |
| --- | --- | --- | --- |
| Arachidonic aid | 1.2478 | 0.0170 | DOWN |
| Myristic acid | 1.3709 | 0.0075 | DOWN |
| 1,2-Di-(9Z-octadecenoyl)-sn-glycero-3-phosphocholine | 1.6114 | 0.0001 | UP |
| 1-Hexadecanoyl-2-octadecadienoyl-sn-glycero-3-phosphocholine | 1.3777 | 0.0044 | UP |
| 1-Hexadecanoyl-2-(9Z,12Z-octadecadienoyl)-sn-glycero-3-phosphoric acid | 1.2339 | 0.0217 | UP |
| 1-Octadecyl-2-acetyl-sn-glycero-3-phosphocholine | 1.5362 | 0.0008 | DOWN |
| 1-Hexadecanoyl-sn-glycero-3-phosphocholine | 1.2082 | 0.0285 | DOWN |
| Sphinganine | 1.0906 | 0.0336 | UP |
| Sphingosine 1-phosphate | 1.1225 | 0.0495 | UP |
| Sphingomyelin | 1.1286 | 0.0403 | UP |
| (S)-Malate | 1.5513 | 0.0005 | UP |
| Citrate | 1.6947 | <0.0001 | UP |
| 2-Oxo-glutarate | 1.4627 | 0.0042 | UP |
| L-Kynurenine | 1.4781 | 0.0026 | UP |
| Formylanthranilic acid | 1.4197 | 0.0038 | UP |
| Indole-2-carboxylic acid | 1.6909 | <0.0001 | UP |
| Phenylalanine | 1.4193 | 0.0036 | UP |
| 3-Phenylpropanoic acid | 1.6626 | <0.0001 | UP |
| 3-(3-Hydroxyphenyl) propanoic acid | 1.6611 | <0.0001 | UP |
| Glycine | 1.5797 | 0.0004 | DOWN |
| Dimethylglycine | 1.6503 | 0.0001 | UP |
| Aspartate | 1.6033 | 0.0003 | UP |
| Glutamate | 1.5163 | 0.0023 | UP |
| Glycochenodeoxycholate | 1.2562 | 0.0138 | UP |
| Chenodeoxycholate | 1.2297 | 0.0355 | UP |
| Tauroursodeoxycholic acid | 1.2056 | 0.0190 | UP |
| Cholic acid | 1.1992 | 0.0403 | UP |
| Taurine | 1.1848 | 0.0319 | UP |
| choline | 1.4686 | 0.0037 | UP |
| Lactate | 1.5086 | 0.0017 | UP |
| cortisol | 1.4602 | 0.0036 | DOWN |
